# A Case of Erysipelatous Pneumonia

**Published:** 1880-08

**Authors:** F. Peterson


### A CASE OF ERYSIPELATOUS PNEUMONIA.

FROM THE FRENCH BY F. PETERSON, M. D.

A LABORER, aged 55, entered hospital Necker, June 19, 1876. Eight days before, this man was taken while at work with a high fever, accompanied by severe chill, vomiting and painful respiration. The fever was persistent the first few days, the appetite was completely lost, the dyspnœa increased, but there was neither viscous expectoration nor pneumonic sputa. Patient felt a well-marked pain at the base of the right lung. The trunk in the neighborhood of the right hypochondriac region became very sensitive to pressure.

The day of entrance: had the stitch in the side; the conjunctiva had a subicteric tint; the liver was somewhat enlarged and slightly sore on pressure; pulse 92, temperature 101.8° Fahr.; no expectoration; anorexia; constipation; cephalalgia; general lassitude.

On percussion of the chest we found behind and at the base of the right lung a dull sound, and an increase of thoracic vibration; in this vicinity the respiration was sighing, and a number of sub-crepitant rales were clearly distinguished; heart sounds normal.

From these symptomatic data, and taking into account the manner of inception and progress of the malady, the following diagnosis was made: Pneumonia of the right base, with some reserves in favor of pulmonary congestion, for there were a few sub-crepitant rales at the left base also, and I thought that this congestion might depend upon a bronchitis, the symptoms of the patient being of a nature to permit such a supposition. There was in effect such a state as one often observes at the end of both pneumonia and bronchitis. Consequently pneumonia in the stage of resolution or bronchitis with pulmonary engorgement were the two hypotheses possible.

During the two days following there were the same general state, and same signs in auscultation. But the third day, in the morning, we discovered an erysipelatous patch upon his nose, which extended very rapidly over the rest of his face, but not accompanied by intense febrile movement. There was no sign of laryngitis or angina. The erysipelas was benign, and some days after the patient was cured of both that and the pulmonary affection.

The appearance of this facial erysipelas leads one to believe that perhaps this was a case of pulmonary erysipelas. The patient then told us that some days before the onset of his disease, he had slept in the same bed previously occupied by one of his daughters affected with erysipelas of the face.

It seems then that this man offers us an example of internal erysipelas, first manifested in the lung, and finally followed by erysipelas of the face. I ought to remark that the erysipelas appeared to have leaped, so to say, from the lung to the face, without invading the intermediary parts, for, as I before observed, there were no phenomena of laryngitis or angina before the appearance of the facial disorder.—*Prof. Potain in "La France Medicale.*"

